# Modulation of Perturbed Cardiac Metabolism in Rats Under High-Altitude Hypoxia by Combination Treatment With L-carnitine and Trimetazidine

**DOI:** 10.3389/fphys.2021.671161

**Published:** 2021-06-28

**Authors:** Hebing Xie, Gang Xu, Jiye Aa, Shuhua Gu, Yuqi Gao

**Affiliations:** ^1^Institute of Medicine and Hygienic Equipment for High Altitude Region, College of High Altitude Military Medicine, Army Medical University (Third Military Medical University), Chongqing, China; ^2^Changzhou Shanmei Pharmaceutical Research and Development Center Co., Ltd., Changzhou, China; ^3^Key Laboratory of Drug Metabolism and Pharmacokinetics, China Pharmaceutical University, Nanjing, China

**Keywords:** acute mountain sickness, L-carnitine, trimetazidine, metabolomics, therapeutic effect, hypoxia, myocardium, cardiomyocytes

## Abstract

High-altitude hypoxia has long been recognized as a vital etiology for high-altitude illnesses. High-altitude myocardial injury (HAMI) usually occurs in people who suffered from high-altitude exposure. To date, the molecular mechanism of HAMI remains elusive, which seriously hinders the prevention and treatment of HAMI. L-carnitine and trimetazidine are classic cardiovascular protective medicines. In this study, we used the metabolomic method, based on GC/MS, to explore the changes in metabolites in rats exposed to high-altitude hypoxia and then illustrate the metabolic pathways associated with the modulatory effect of L-carnitine combined with trimetazidine on rats with high-altitude exposure. The results showed that metabolites in the myocardium in rats under high-altitude hypoxia were markedly changed, such as branched-chain amino acids (BCAA, leucine, isoleucine, and valine), taurine, succinic acid, fumaric acid, lactic acid, pyruvic acid, 3-hydroxybutyrate, and docosahexaenoic acid (DHA), while L-carnitine combined with trimetazidine modulated and improved the abnormal changes in energy substances caused by high-altitude hypoxia. L-carnitine mainly promoted the metabolism of fatty acids, while trimetazidine enhanced the glycolysis process. The combined administration of the two components not only increased the metabolism of fatty acids but also promoted aerobic glycolysis. Meanwhile, it contributed to the decrease in the elevation in some of the intermediates of the tricarboxylic acid (TCA) cycle, decrease in the production of 3-hydroxybutyric acid, and relief of the abnormal energy metabolism process in organisms and the cardiac tissue. Our analysis delineates the landscape of the metabolites in the myocardial tissue of rats that were exposed to high altitude. Moreover, L-carnitine combined with trimetazidine can relieve the HAMI through modulated and improved abnormal changes in energy substances caused by high-altitude hypoxia.

## Introduction

A considerable proportion of people who live in plains will suffer from high-altitude illnesses when they suddenly ascend to an altitude of over 2,500 m without acclimatization, such as acute mountain sickness (AMS), high-altitude pulmonary edema (HAPE), and high-altitude cerebral edema (HACE; Aksel et al., 2019). Moreover, multiple physiologic changes occur during exposure to hypoxia for acclimatization that impacts the cardiovascular system. Overcompensation or decompensation induces high-altitude hypoxia and leads to cardiac diseases easily, mainly leading to cardiac failure and cardiac arrhythmia. High-altitude myocardial injury (HAMI) usually occurs during the process (Donegani et al., 2014). There are no studies on the molecular mechanisms of HAMI by far, and its prevention and control need immediate attention.

L-Carnitine is an endogenous molecule involved in fatty acid metabolism. Its main function is to promote lipid metabolism. L-carnitine transports the long-chain fatty acid into the mitochondrial matrix, thereby delivering substrate for oxidation and subsequent energy productions (Pekala et al., 2011; Adeva-Andany et al., 2017). In addition, L-carnitine is associated with increased cytochrome c reductase of reduced nicotinamide adenine nucleotide (NADH) and enhanced cytochrome oxidase activity, thereby accelerating ATP production (Huertas et al., 1992). For ischemia and hypoxia of a variety of tissues, L-carnitine can enhance energy supplies to tissues and organs by increasing energy (Wang et al., 2018). As a well-established anti-angina agent, trimetazidine hydrochloride promotes the oxidation of carbohydrates at the cellular level, provides protection for metabolic myocardium, and demonstrates robust efficacy on ischemic cardiac injury (Dezsi, 2016; Marzilli et al., 2019). There have been a large number of studies reporting the efficacy and mechanism of action underlying L-carnitine and trimetazidine hydrochloride and also studies on the efficacy of the combination of L-carnitine and trimetazidine for the treatment of pump failure after acute myocardial infarction in clinical practice (Fragasso et al., 2008; Zhang et al., 2019). However, the efficacy of such a combination on the prevention and treatment of HAMI remains elusive.

Metabolomics is defined as systematic studies that quantitatively detect the dynamic multiparametric metabolic responses of living systems to genetic modification or pathophysiological stimuli. It can provide a unique perspective for the complex and dynamic metabolic changes in organisms under the disease state or drug intervention (Johnson et al., 2016). So, we analyzed metabolic changes of cardiomyocytes in rats under high-altitude hypoxia induced by the hypobaric chamber–simulated high-altitude environment; the effects of modulation of L-carnitine combined with trimetazidine on metabolism and energy changes in cardiomyocytes in rats to illustrate the metabolite landscape of the myocardial tissue after rats were exposed to high altitude; and the mechanisms of L-carnitine combined with trimetazidine in the prevention of HAMI.

## Materials and Methods

### Animals

Healthy male Sprague–Dawley (SD) rats weighing 200 ± 20 g were provided by the Experimental Animal Center of the Third Military Medical University (license number: SYXK2012-0030, production license number: SCXK 20120011). The animals were acclimated in the polypropylene cages (at most three rats per cage) for 3 days and given ordinary feeds, with *ad libitum* access to drinking water. Other conditions included a 12-h light/dark cycle (lights on from 6:00 a.m. to 6:00 p.m., and off from 6:00 p.m. to 6:00 a.m.) with an indoor temperature of 22–24°C and relative humidity of 45 ± 5%. All operations on animals were performed under the guidance of the Animal Research Committee and approved by the Animal Ethics Committee of the Institute.

### Materials and Reagents

L-carnitine was purchased from Northeast Pharmaceutical Group Co., Ltd. (production batch No: 017140401) and trimetazidine hydrochloride was purchased from Suzhou pharmaceutical factory, Jiangsu Wuzhong Pharmaceutical Group Co., Ltd. (production batch No: Y1H130802). The 99 atom%^13^C, Myristic-1,2-^13^C_2_ acid, methoxyamine hydrochloride (purity of 98%), the stable isotope-labeled internal standard compound (IS), pyridine (≥99.8% GC), and standard alkane solution (C8–C40) were obtained from Sigma–Aldrich. About 1% of trimethylchlorosilane (TMCS) and MSTFA (*N*-methyl-*N*-trimethylsilyl trifluoroacetamide) were acquired from Pierce Chemical Company (Rockford, United States). N-heptane and methanol were of HPLC grade and purchased from Merck (Darmstadt, Germany) and the Tedia Company (Fairfield, United States), respectively. Besides, the purified water was from the Milli-Q system (Millipore, Bedford, United States). A DYCQ-simulated high-altitude low-pressure chamber was jointly developed by our laboratory and Guizhou Fenglei Aviation Ordinance Co., Ltd. (Guizhou, China).

### Animal Model and the Doses

After 3 days of adaptive feeding, the experimental animals were randomly assigned to five groups: (1) control group; (2) HAMI model group (Model); (3) L-carnitine and trimetazidine pretreatment group (LT), administered intragastrically by 400 mg/kg/day of L-carnitine and 2 mg/kg/day of trimetazidine; (4) l-carnitine pretreatment group (L), administered intragastrically by 400 mg/kg/day of L-carnitine; and (5) trimetazidine pretreatment group, administered intragastrically by 2 mg/kg/day of trimetazidine (T). The rats were pre-administered with the corresponding drugs at 5 ml/kg two times daily for a total of 7 days, and rats in the plain control group and the model control group were given the corresponding normal saline. After the experimental animals were transferred to the hypobaric chamber (Guizhou Fenglei Aviation Ordinance Co., Ltd), the chamber was depressurized at 6 m/s to simulate an altitude of 7,000 m. The altitude was reduced to 5,000 m at the time of administration and sampling, and the administration was continued in accordance with the original dosing regimen in the hypobaric chamber; 48 h after entering the low-pressure chamber, the rats were subjected to anesthesia through an intraperitoneal injection of 10% urethane at 1.5 g/kg, with placement in the supine position for fixation. The cardiac tissue was harvested quickly (with the connective tissue removed), cut open, and placed in normal saline for the removal of blood. After being blotted with filter paper, the cardiac tissue was wrapped with tin foils and preserved in liquid nitrogen at −80°C.

### Cell Culture

A 2- to 3-day-old SD rat was immersed in 75% alcohol for 1 min, and then the heart was taken out and put into precooled D-Hank’s solution. The blood clot and fibrous tissue around the heart were removed. The heart was placed in another small beaker containing D-Hank’s solution and cut into pieces of about l mm^3^. About 0.25% of trypsin and 3 ml of 0.3 g/L type II collagenase was added to the beaker and digested at 37°C for 10 min, and the complete medium was then added to stop digestion. After filtration with 200 mesh screen and centrifugation at 1500 rpm, the precipitates were inoculated in Dulbecco’s modified Eagle’s medium (DMEM) complete medium containing 1% penicillin–streptomycin and 10% FBS and cultured at 37°C and 5% CO_2_ saturation humidity for 1.5 h, and then, the nonadherent cells were collected. After centrifugation and counting, the cardiomyocytes were randomly seeded in a 24-well plate and cultured in DMEM in an incubator containing 5% CO_2_ at 37°C. The cardiomyocytes were assigned into five groups: (1) normoxia culture group; (2) hypoxia culture group; (3) low-dose group, which was cultured in the medium containing 2 μg of L-carnitine and 0.01 μg/ml of trimetazidine hydrochloride; (4) median-dose group, which was cultured in the medium containing 4 μg of L-carnitine and 0.02 μg per ml of trimetazidine hydrochloride 0; and (5) high-dose group, which was cultured in the medium containing 8 μg of L-carnitine and 0.04 μg per ml of trimetazidine hydrochloride. Each group had three wells. After culturing for 5 days, the medium was changed to DMEM without FBS. Then, the cells in the normoxia culture group were cultured in the incubator with a normal oxygen concentration of 21%. The cells in the hypoxia culture group and drug pretreatment groups were put into an incubator with 5% CO_2_ and 1% O_2_ for 5.5 h.

### Cell Viability Assay

For determination of cell viability, cardiomyocytes exposed to hypoxia and treated by L-carnitine combined with trimetazidine or the normoxia group were plated into a flat bottom 96-well plate at 4 × 10^3^ cells per well in triplicate with 100 μl medium. Ten microliter of Cell Counting Kit-8 reagent (CCK-8; Dojindo) was added to each well. After incubation for 2 h, the optical density value at the wavelength of 450 nm was measured using a microplate reader (Bio-Rad Laboratories, Inc.).

### Cell Apoptosis Assay

Cell apoptosis was detected using the Annexin V-FITC Apoptosis Detection Kit (Calbiochem/EMD/Merck KGaA) according to the instructions of the manufacturer.

### Sample Preparation for Metabolomics Study

For the GC/MS analysis, myocardial tissue samples were subjected to pretreatment, extraction, and derivatization as previously reported (Zhu et al., 2020). Profiling of the endogenous molecules in the tissues was conducted using Shimadzu GCMSQP2010 (Shimadzu Corp., Tokyo, Japan), which was equipped with an RTx-5MS column (30 m × 0.25 mm i.d. fused-silica capillary column chemically bonded with a 0.25 μm cross bond, 5% diphenyl/95% dimethyl polysiloxane, Restek Corporation, PA, United States).

Identification of metabolites was carried out through comparison of the mass spectra (MS) and the retention indices of the detected compounds with the reference standards and those from the databases below: Wiley 9 (Wiley–VCH Verlag GmbH & Co. KGaA, Weinheim, Germany), the National Institute of Standards and Technology (NIST) library 2.0 (2008), and an in-house mass spectra library database, which was established by the Umea° Plant Science Center, Swedish University of Agricultural Sciences (Umea°, Sweden). Following identification, one feature ion was selected as the quant mass, and the peak area was acquired for each peak/compound, respectively.

### Multivariate Data Statistical Analysis

For each detected peak, the relative quantitative peak areas were normalized to myristic-1,2-^13^C_2_ acid, the stable isotope IS, before the adoption of SIMCA-P 13 software (Umetrics, Umea°, Sweden) for multivariate statistical analysis. An evaluation of the impact of Adriamycin on metabolic pathways was performed based on a tool for metabolomic data analysis available online.^1^ The Pathway Analysis module represented a combination of the results from powerful pathway enrichment analysis and those from the pathway topology analysis so as to facilitate the identification of the most relevant pathways involved in the study conditions for researchers. The built-in *Rattus norvegicus* (rat) pathway library for pathway analysis and hypergeometric test for over-representation analysis was employed by uploading the discriminatory compounds. A report on the results was then presented in the form of graphs as well as detailed tables. Potential drug efficacy and/or resistance biomarkers were identified on the basis of established metabolic pathways and statistics.

### Statistical Analysis

Data were presented as mean ± SD of at least three independent experiments. The differences between the two means were analyzed with the Student’s *t*-test using the software Graphpad Prism 6.0 (California, United States). Multiple experimental groups were analyzed by a one-way ANOVA followed by Tukey’s *post-hoc* test to obtain statistical differences. A value of *p* < 0.05 indicated a statistically significant difference.

## Results

### L-carnitine Combined With Trimetazidine Alleviates Hypoxia-Induced Cardiomyocyte Apoptosis

**Figure 1:**
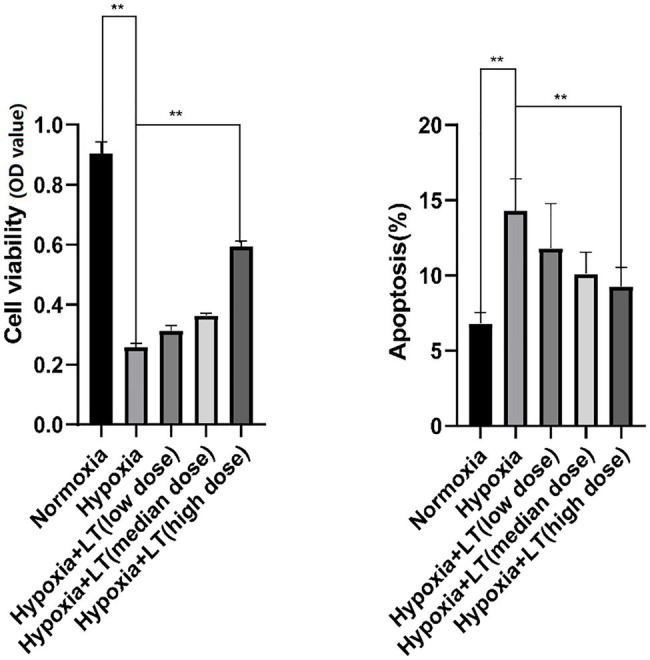
Effects of L-carnitine combined with trimetazidine on cardiomyocytes cultured by hypoxia. Cells were pretreated with or without L-carnitine and trimetazidine (LT), and then cultured in a hypoxic condition. Cells constantly cultured in a hypoxic condition were considered as the hypoxia group. Cells constantly cultured in a normoxic condition were considered as the normoxia group. Cell viability was determined using CCK-8 assay. Cell apoptosis was detected using an Annexin V-FITC apoptosis detection kit. ^**^p < 0.01.

To ascertain the effects of L-carnitine combined with trimetazidine on hypoxia-induced injury in cardiomyoblasts, myocardial cells were treated with gradient dilutions of L-carnitine and trimetazidine hydrochloride before exposure to hypoxia. Results of the CCK-8 assay indicated that cell viability was significantly lower in the cells exposed to hypoxia when compared with the cells not exposed to hypoxia. It was also observed that cell viability was obviously higher in the cells treated with the high-dose compound, L-carnitine, than that in cells exposed to hypoxia (Figure 1). These data demonstrated that exposure to hypoxia decreased cell survival, while L-carnitine combined with trimetazidine protected myocardial cells from hypoxia-induced decrease in cell survival in a dose-dependent manner. Furthermore, cell apoptosis was detected by PI/Annexin V staining assay (Figure 1). The results revealed that exposure to hypoxia significantly induced cardiomyocyte apoptosis. Notably, L-carnitine combined with trimetazidine significantly alleviates hypoxia-induced myocardial apoptosis.

### GC/MS Profiles of Metabolites in the Cardiac Tissue and Overview of the Metabolomic Data

**Figure 2:**
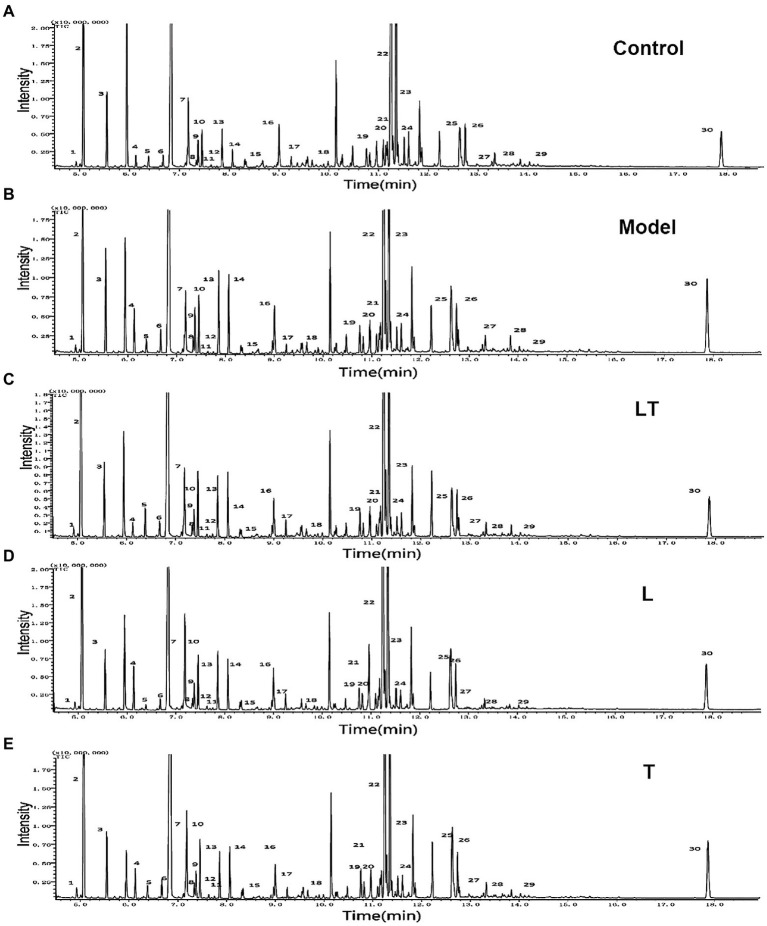
Typical GC/MS chromatograms of cardiac tissue extracts in **(A)** the control group; **(B)** the high-altitude myocardial injury (HAMI) model group; **(C)** the LT group; **(D)** the L-carnitine group; and **(E)** the trimetazidine group. Visual inspection of the chromatograms revealed obvious differences between the groups. L: L-carnitine; T: trimetazidine; LT: L-carnitine combined with trimetazidine. The compounds were identified as 1. pyruvate; 2. lactate; 3. alanine; 4. 3-hydroxybutyrate; 5. urea; 6. valine; 7. leucine; 8. isoleucine; 9. proline; 10. glycerate; 11. fumarate; 12. serine; 13. succinate; 14. malate; 15. 2-ketoglutaric acid; 16. asparagine; 17. taurine; 18. citrate; 19. fructose; 20. glucose; 21. lysine; 22. tyrosine; 23. octadecadienoate; 24. oleic acid; 25. tryptophan; 26. arachidonic acid; 27. D-mannose-6-phosphate; 28. docosahexaenoic acid; 29. D-galactofuranose; and 30. cholesterol.

**Figure 3:**
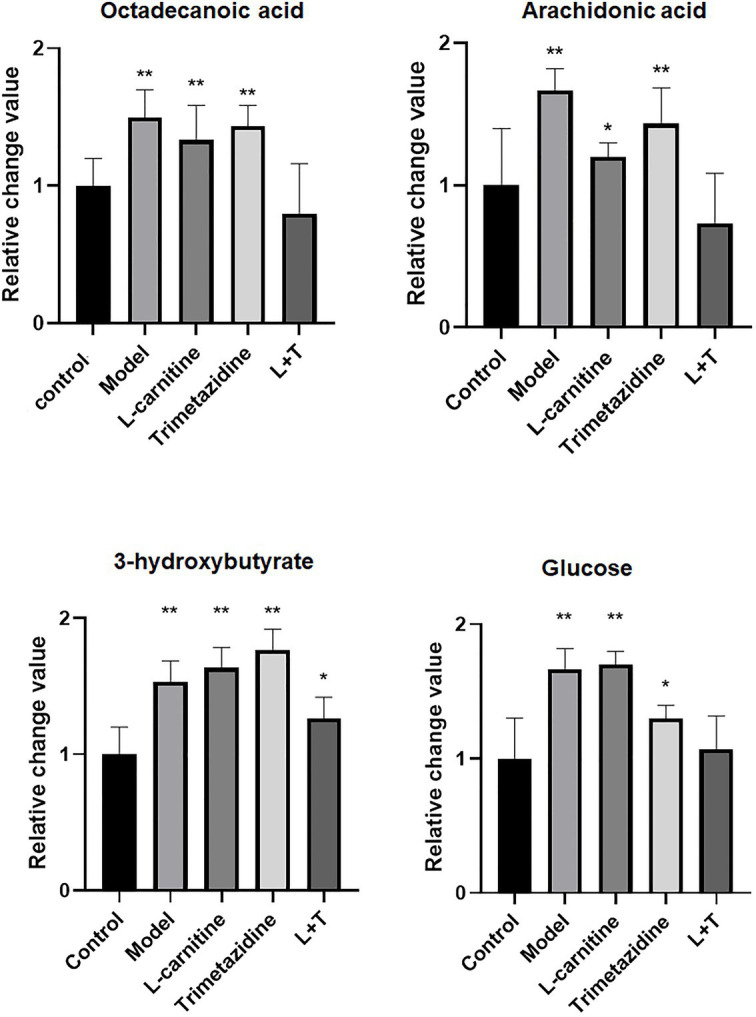
A quantitative map of comparison of key metabolites in the myocardial tissue. LT represents L-carnitine combined with the trimetazidine group. *p < 0.05 and **p < 0.01 compared with the control group.

After treatment with L-carnitine, trimetazidine, and L-carnitine combined with trimetazidine, GC/MS was used to detect the content of various metabolites in the myocardium of rats with high-altitude hypoxia, and the metabolic spectrum information was collated. A typical mass spectrogram of total ion flow is shown in Figure 2. Significant differences between the animals in the model group and the control group were indicated *via* visual inspection of the GC/MS profiles of metabolites in the cardiac tissues. A total of 111 peaks in cardiac tissue were resolved through deconvolution of the chromatograms, among which 83 metabolites were authentically identified. These metabolites included amino acids, fatty acids, lipids, carbohydrates, and purines (Supplementary Table S1). The relative change value of the key metabolites (octadecanoic acid, arachidonic acid, 3-hydroxybutyrate, and glucose) in the metabolism of some fatty acids, ketones, and glucose is shown in Figure 3.

**Figure 4:**
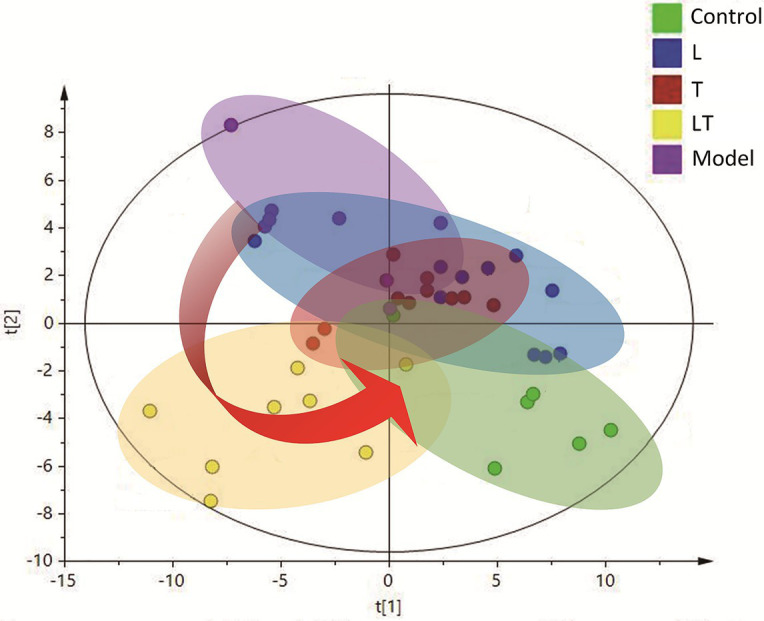
The Partial Least-Squares Discriminant Analysis (PLS-DA) scores plot of the HAMI model rats and the rats treated. The green plots are the control group; the blue plots are the L-carnitine group (L); the red plots are the trimetazidine group (T); the yellow plots are the LT group; and the purple plots are the HAMI model group.

In order to acquire the quantitative data, a quant mass was selected for each molecule and the peak area was obtained for each peak/molecule convoluted. An unsupervised PCA model, on the basis of the data matrix, was adopted for an overview of the data set. The scores plot revealed that the samples in the same group showed a tendency of clustering closely, but the samples from different groups were scattered to varying degrees. An unsupervised PCA model was, on the basis of the data matrix, adopted for an overview of the data set. No outliers were noted in the PCA model. The Partial Least-Squares Discriminant Analysis (PLS-DA) plots demonstrated that induction for 48 h by high-altitude hypoxia significantly deviated the plots from the control, indicating that the metabolites in the cardiac tissue were strongly disturbed by high-altitude hypoxia (Figure 4). The PLS-DA models explicitly suggested that L-carnitine combined with trimetazidine has a high-altitude hypoxia-induced deviating tendency and that, compared to the other groups, samples treated with L-carnitine combined with trimetazidine showed a tendency of moving closer to the controls, indicating a recovery of metabolic disorders have a tendency to return to control levels (Figure 4).

### High-Altitude Hypoxia-Induced Perturbed Metabolites in the Cardiac Tissue

**Figure 5:**
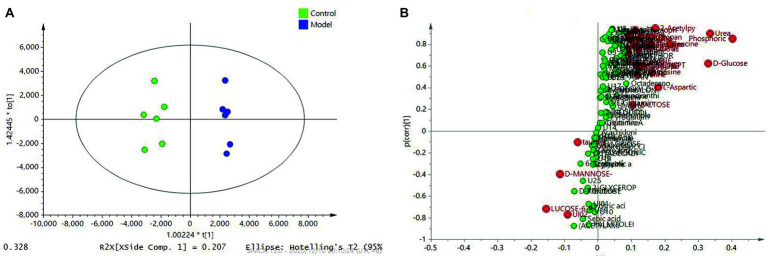
PLS-DA analysis revealed the different metabolic patterns and the discriminant metabolites induced by the HAMI model and normal control based on metabolites in the myocardial tissue. **(A)** PLS-DA between the model and the control; **(B)** S-plot analysis of metabolites differentiating the model and the control.

With the aim of screening out the variable of difference from a large number of data, the biological information of significance was obtained. To this end, we established reliable and stable mathematical models to guide and identify the metabolites in the myocardial tissue, as well as the effects of drug-induced metabolic and biochemical pathways, biomarker, and potential pharmacodynamics mechanisms at altitude hypoxia. We selected mass-to-charge ratio (*m*/*z*) to conduct quantitative analysis of the obtained peaks and obtained the peak area corresponding to endogenous substances. The peak areas of all compounds in the model group and the administration group were compared with the control group, and a double-tailed statistical *t*-test was conducted. The statistical t-test with *p* ≤ 0.05 showed a significant difference in metabolic substances between the model group and the administration and control group. The PLS-DA supervisory model was applied to analyze the peak area data corresponding to the endogenous substances obtained by SIMCA 14.0 software, and PLS-DA highlighted the differences between the two groups of samples. Compared with the control group, the PLS-DA model showed good typing (Figure 5A). Through comparison and analysis of the s-plot model, the credibility of the contribution of metabolic changes to the model can be more intuitively observed, as shown in Figure 5B. In this model, the *x*-coordinate is the credibility (correlation) and the *y*-coordinate is the contribution (covariance). The greater the weight distributed at both ends of “S,” the greater was the difference.

Metabolic impact analysis indicated high-altitude hypoxia to distinct perturbation of metabolism that involved amino acids, carbohydrates, TCA intermediates, free fatty acids, etc., in accordance with the discriminant metabolites confirmed in the cardiac tissue. Specifically, high-altitude hypoxia led to elevated levels of carbohydrates (glucose, fructose, galactose, and mannose), while its downstream product, pyruvic acid, was elevated as well. Meanwhile, there was a significant elevation of free fatty acids, TCA intermediates (citrate, malate, fumarate, and 2-ketoglutarate), and 3-hydroxybutyrate in the cardiac tissue of the animal models. The increment in the level of ornithine and urea suggested that the transamination of amino acids was enhanced, while the rise in urea indicated abnormal purine metabolism (Table 1).

**Table 1:** The perturbed cardiac tissue metabolites induced by high-altitude hypoxia on the metabolites.

Metabolic pathways/metabolites	The regulatory tendency and statistical analysis (HAMI model group vs. Control group)	
**Amino acid metabolism**		
Alanine, proline, serine, threonine, tryptophan, asparagine, lysine, and tyrosine	↑	∗
Taurine	↓	∗
**Branched chain amino acid**		
Valine, leucine, and isoleucine	↑	∗
**Fatty acids**		
Oleic acid	±	/
Octadecanoic acid, arachidonic acid, and docosahexaenoic acid	↑	∗
Glyceric acid	±	/
**Ketone**		
3-Hydroxybutyrate	↑	∗
**Carbohydrates and glycolysis**		
Glucose, fructose, galactose, and mannose	↑	∗
Lactate	↑	/
Pyruvate	↑	/
**TCAs**		
Citrate	↑	/
Malate	↑	∗
Fumaric acid	±	/
α-Ketoglutaric acid	↑	∗
Succinic acid	±	/

↓: downregulated by at least 20%, respectively; ↑: upregulated by at least 20%, respectively; ±: marginally regulated without statistical significance. ^∗^*p* < 0.05 (one-way ANOVA); /: *p* > 0.05 (one-way ANOVA).

### Modulation of Perturbed Metabolites in the Cardiac Tissue by L-carnitine Combined With Trimetazidine

**Figure 6:**
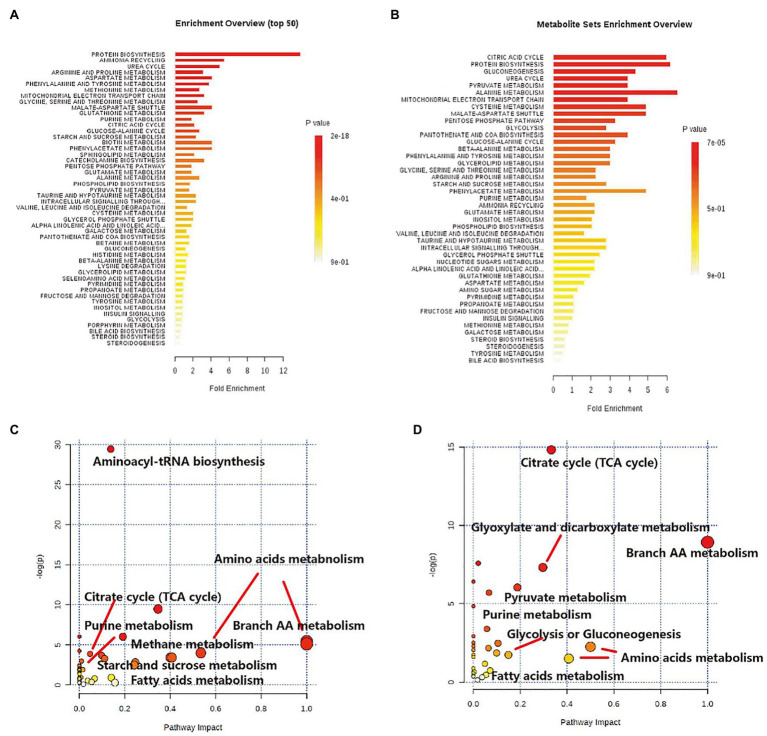
The impact analysis shows the effect of high-altitude hypoxia and LT on primary metabolic pathways based on cardiac tissue metabolome. **(A)** Enrichment analysis of metabolic sets induced by L-carnitine, relative to model control. **(B)** Enrichment analysis of metabolic sets modulated by trimetazidine, relative to model control. **(C)** Impact analysis of metabolic sets modulated by L-carnitine, relative to model control. **(D)** Impact analysis of metabolic sets modulated by trimetazidine, relative to model control.

Metabolomics studies of the myocardial tissue on L-carnitine and trimetazidine individually demonstrated that L-carnitine increased the level of 3-hydroxybutyric acid. Besides, L-carnitine affected the metabolism of fatty acids, pyruvate, amino acids, and branch amino acids (Supplementary Table S2). Trimetazidine decreased the level of glucose significantly. However, trimetazidine significantly affected the levels of TCA intermediates and substances associated with TCA (Supplementary Table S2). The enrichment analysis also proved this point (Figure 6). As shown clearly in Figures 6A,C, L-carnitine mainly modulated the metabolism of fatty acids, amino acids, and pyruvate. Trimetazidine mainly modulated the metabolism of amino acids, TCA intermediates, and carbohydrates. Interestingly, both L-carnitine and trimetazidine significantly downregulated the content of amino acids in the myocardial tissue, while L-carnitine combined with trimetazidine had no effects on most amino acids. Instead, it significantly upregulated some amino acids, such as branched amino acids (Supplementary Table S2).

**Figure 7:**
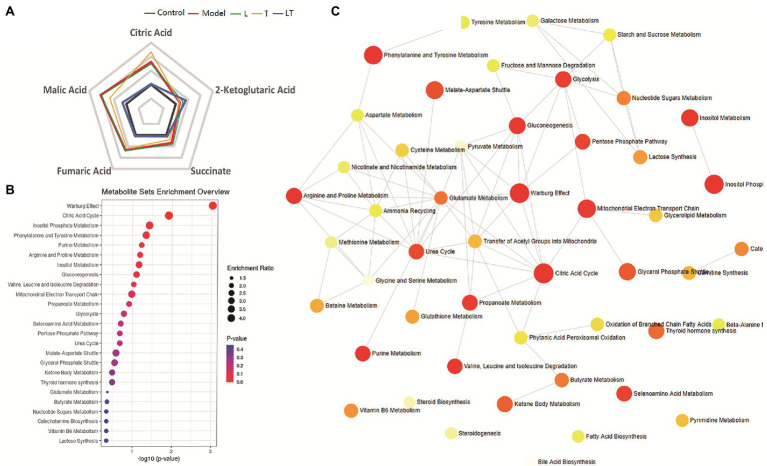
The effect of the regulatory effect of L-carnitine combined with trimetazidine on five key molecules and primary metabolic pathways based on the metabolome of cardiac tissue. **(A)** A phase diagram for the five key molecules of cardiac tissue involved in glycolysis, tricarboxylic acid (TCA), and fatty acid metabolism. **(B)** Metabolite sets enrichment overview. **(C)** Metabolic network pathway of L-carnitine combined with trimetazidine by HAMI model based on metabolites in the myocardial tissue. L: L-carnitine; T: trimetazidine; LT: L-carnitine combined with trimetazidine.

Treatment with L-carnitine combined with trimetazidine significantly reversed elevations in fatty acids and TCA intermediates due to high-altitude hypoxia and meanwhile reduced the contents of lactic acid and pyruvate (Supplementary Table S2). Based on the pivotal molecules associated with energy metabolism in the myocardial tissue, a radar map was established that showed the changes in the energy metabolites in the cardiac under high-altitude hypoxia as well as the modulation of the metabolites by L-carnitine, trimetazidine, and L-carnitine combined with trimetazidine (Figure 7A). And metabolic impact analysis indicated that treatment with L-carnitine combined with trimetazidine can perturb the metabolism of amino acids, branched-chain amino acids, carbohydrates, and TCA intermediates (Figure 7B). Table 1 shows that high-altitude hypoxia induced a deviation in the TCA cycle and free fatty acids metabolism, especially for the pivotal molecules of 3-hydroxybutyrate, while L-carnitine combined with trimetazidine partially rectified the metabolic disturbance. The modulatory effects of L-carnitine combined with trimetazidine balanced the glycolysis and oxidation of fatty acids, and the major effects involved in metabolic pathways are Warburg effect, the TCA cycle, inositol phosphate metabolism, phenylalanine and tyrosine metabolism, purine metabolism, arginine and proline metabolism, glycol metabolism, branched-chain amino acid degradation, and fatty acids metabolism (Figures 6B,D). Analysis of metabolic network pathway also corroborates these conclusions (Figure 7C). In a word, L-carnitine combined with trimetazidine corrected the disorder of energy metabolites in rats. These interesting results provide a very big hint for the study of the pharmacological mechanism of drugs against altitude hypoxia.

## Discussion

To date, there has been a lack of understanding of the effects of high-altitude hypoxia on the myocardial tissue and its metabolic characteristics. Moreover, the metabolic pathways involved in the treatment of L-carnitine combined with trimetazidine on high-altitude–exposed rats remain elusive. This study demonstrated that the substances associated with energy metabolism in the myocardial tissue under high-altitude hypoxia were significantly changed. L-carnitine combined with trimetazidine modulated and improved the abnormal changes in energy substances caused by high-altitude hypoxia. L-carnitine mainly promoted the metabolism of fatty acids, amino acids, and carbohydrates, while trimetazidine mainly enhanced the metabolism of amino acids and carbohydrates.

High-altitude hypoxia contributed to an insufficient systemic oxygen supply, and the relevant energy metabolism was changed accordingly (Murray, 2016). The marked decrease in the content of fatty acids in the blood suggested that fatty acid oxidation in organisms was accelerated for organisms to make better use of limited oxygen and meet the needs for energy (Calder, 2015). The significant increase in amino acids *in vivo* demonstrated that the proteins were mobilized and decomposed. The significantly increased 3-hydroxybutyric acid showed that the β-oxidation pathway in the liver was activated, that the level of fatty acids was decreased, and that 3-hydroxybutyric acid as a ketone body was produced in large amounts in the liver mitochondria after fatty acid degradation and entered the peripheral blood as alternative fuels to be supplied to other hypoxic tissues, such as the cardiac. The elevation in fatty acids and TCA intermediates in the myocardium under high-altitude hypoxia indicated a weakened activity of TCA and reduced production of ATP. This might lead to the accumulation of acetyl-CoA and NADH (Adeva-Andany et al., 2019), thereby inhibiting β-oxidation and preventing fatty acid oxidation in cardiomyocytes. Under high-altitude hypoxia, the content of glucose in cardiomyocytes was increased markedly, suggesting an increased uptake of carbohydrates by cardiomyocytes under high-altitude hypoxia.

Widely distributed in different tissues and cells in organisms, L-carnitine is an indispensable cofactor for β-oxidation of fatty acids after they enter the mitochondria. It can promote the normal operation of the TCA cycle and help cells maintain the energy generation necessary for their physiological activities (Walter, 1996). L-carnitine is the substrate of carnitine acetyltransferase (CrAT). When acetyl-CoA accumulates in mitochondria, the enzyme converts the accumulated acetyl-CoA to acetyl carnitine, reduces the acetyl-CoA/CoA ratio in mitochondria, promotes the activity of pyruvate dehydrogenase complex, and promotes the oxidative decarboxylation of pyruvate to acetyl-CoA (Calvani et al., 2000). This study demonstrated that, after treatment with L-carnitine alone, in the myocardial tissue, L-carnitine mainly contributed to the reversal of most amino acids (including branched chain amino acids) and some of the free fatty acids (including DHA and arachidonic acid) caused by high-altitude hypoxia as well as the decreased level of pyruvate. However, it did not change the abnormality in taurine, glucose, lactate, TCA intermediates, and 3-hydroxybutyric acid, suggesting that the administration of L-carnitine alone enhanced the fatty acid β-oxidation and pyruvate oxidation. Pyruvate is an intermediate product of aerobic glycolysis. Oxidative decarboxylation of pyruvate to acetyl-CoA is a key step in aerobic glycolysis. Thus, L-carnitine enhances aerobic glycolysis by promoting the metabolism of pyruvate.

Trimetazidine, one of the novel metabolite cytoprotection belonging to the piperazine class, demonstrates effects in protecting mitochondria (Dedkova et al., 2013), mitigating intracellular acidosis (Stadnik et al., 2013) and calcium overload (Wei et al., 2015), scavenging oxygen free radicals, and combating oxidation (Maridonneau-Parini, 1986). It can prevent the decrease in ATP within cells by protecting the energy metabolism within ischemic cells, and its protective effect on hypoxic myocardium has been extensively used in the treatment of coronary cardiac diseases and myocardial ischemia. This study revealed that, after treatment with low-dose trimetazidine alone, the level of some of the carbohydrates and amino acids was reversed, and the level of TCA intermediates was further increased, indicating that trimetazidine might result in the reduction of fatty acid oxidation and decomposition and metabolism of proteins in organisms. Trimetazidine significantly downregulated carbohydrates, such as glucose, fructose, galactose, and mannose, indicating that the administration of trimetazidine strengthened the metabolism of carbohydrates in the myocardium and enhanced the glycolysis process.

It was surprising and creative that L-carnitine and trimetazidine hydrochloride were combined at a ratio of 200:1. After treatment with L-carnitine combined with trimetazidine, 3-hydroxybutyrate was significantly decreased. The significant decrease in arachidonic acid, octadecadienoic acid, DHA, glycerophosphate, and 3-hydroxybutyrate in cardiomyocytes indicated that L-carnitine combined with trimetazidine affected the metabolism process of lipids and promoted oxidation and metabolism of fatty acids, as well as the production of ATP, and reduced the accumulation of fatty acids and the use of 3-hydroxybutyric acid. The significant decline in glucose and mannose, as well as lactic acid and pyruvate in cardiomyocytes, indicated that L-carnitine combined with trimetazidine affected the glycolysis process, enhanced aerobic glycolysis, and attenuated the anaerobic glycolysis.

To sum up, high-altitude hypoxia resulted in a relatively huge difference from the normal group with respect to a large amount of endogenous substances in the myocardial sample. The changes in these small endogenous molecules, especially energy substances, suggested that organisms might produce the stress response owing to high-altitude hypoxia; that protein mobilization and decomposition were enhanced; that ketone bodies as alternative energy substances were produced in a large amount *via* fatty acid β-oxidation; and that the anaerobic glycolysis was strengthened. In the myocardium, fatty acid oxidation was inhibited, the glycolysis process was affected, the increase and circulation of TCA intermediates were in a poor status (the TCA metabolite ascending cycle is not smooth), and ATP production was decreased. Therefore, abnormal changes occurred in the entire energy metabolism. L-carnitine combined with trimetazidine modulated and improved the abnormal changes in energy substances caused by high-altitude hypoxia. One of its main components, L-carnitine, mainly promoted the metabolism of fatty acids and aerobic glycolysis, while trimetazidine, another component, enhanced the glycolysis process. Therefore, L-carnitine and trimetazidine have the synergistic effect of promoting aerobic glycolysis. The combined administration of L-carnitine and trimetazidine hydrochloride not only increased the metabolism of fatty acids but also promoted aerobic glycolysis. Meanwhile, it decreased the elevation in some of the TCA intermediates and the production of 3-hydroxybutyric acid and attenuated the abnormal energy generation and the metabolism process in organisms and the myocardial tissue.

## Conclusion

In this study, we first illustrated the metabolite landscape of the myocardial tissue of rats exposed to high-altitude hypoxia. Moreover, we found that L-carnitine combined with trimetazidine could protect the myocardial cells from hypoxia-induced injury through increased metabolism of fatty acids and promoted aerobic glycolysis, providing a new way of thinking for the prevention and treatment of the resulting complications.

## Data Availability Statement

The original contributions presented in the study are included in the article/**Supplementary Material**, further inquiries can be directed to the corresponding author.

## Ethics Statement

The animal study was reviewed and approved by Experimental Animal Center of the Third Military Medical University.

## Author Contributions

HX and YG participated in the design of the study. HX and GX performed the experiments. HX and JA analyzed the data. HX, SG, and YG contributed to the writing of the manuscript. All authors contributed to the article and approved the submitted version.

### Conflict of Interest

HX and SG was employed by company Changzhou Shanmei Pharmaceutical Research and Development Center Co., Ltd.The remaining authors declare that the research was conducted in the absence of any commercial or financial relationships that could be construed as a potential conflict of interest.

## Footnotes

^1^http://www.metaboanalyst.ca
