# Supplementary material for: Modulation of Perturbed Cardiac Metabolism in Rats Under High-Altitude Hypoxia by Combination Treatment With L-carnitine and Trimetazidine
Source: Front Physiol. 2021 Jun 28;12:671161. doi: 10.3389/fphys.2021.671161 (PMC8273437; doi:10.3389/fphys.2021.671161)
Supplement: Supplementary file 1 [file Data_Sheet_1.docx]

Supporting materials-Tables for manuscript:

**Modulation of perturbed cardiac metabolism in rats under high-altitude hypoxia by L-carnitine combined with Trimetazidine**

Table S1. The identified endogenous compounds in cardiac tissue

Table S2. The perturbed cardiac tissue metabolites induced by high-altitude hypoxia and the regulatory effect of drugs on the metabolites

**Table S1. The identified endogenous compounds in cardiac tissue**

| **Identified compounds** | **RT** | **Derivatives** |
| --- | --- | --- |
| 3-Hydroxypyridine | 4.71 | TMS |
| Pyruvate | 4.875 | MEOX, TMS |
| Lactate | 5.015 | 2TMS |
| Diethanolamine | 5.082 | 2TMS |
| Alanine | 5.485 | 2TMS |
| Oxalic acid 1 | 5.88 | 2TMS |
| 3-Hydroxybutyrate | 6.065 | 2TMS |
| Oxalic acid 2 | 6.15 | 2TMS |
| Urea 1 | 6.31 | 2TMS |
| Valine | 6.6 | 2TMS |
| Urea 2 | 6.75 | 2TMS |
| Leucine | 7.065 | 2TMS |
| Glycero l | 7.09 | 3TMS |
| Phosphate 1 | 7.1 | 3TMS |
| Isoleucine | 7.255 | 2TMS |
| Proline | 7.295 | 2TMS |
| Glycine | 7.37 | 3TMS |
| Glycerate | 7.55 | 3TMS |
| Uracil | 7.56 | 2TMS |
| Fumarate | 7.605 | 2TMS |
| Phosphate 2 | 7.645 | 3TMS |
| Pelargonic acid | 7.71 | TMS |
| Serine | 7.77 | 3TMS |
| Theronine | 7.975 | 3TMS |
| 2-Hydroxybutyric acid | 8.125 | 2TMS |
| Beta-Alanine | 8.245 | 2TMS |
| Aminomalonate | 8.55 | 3TMS |
| Succinate | 8.59 | 2TMS |
| Malate | 8.655 | 3TMS |
| 2-Ketoglutaric acid 1 | 8.755 | TMS |
| Aspartate | 8.865 | 3TMS |
| Pyroglutamate | 8.9 | 2TMS |
| 6-Azathymine | 9.025 | 2TMS |
| Cysteine | 9.1 | 3TMS |
| Creatinine | 9.145 | 3TMS |
| 2-Ketoglutaric Acid 2 | 9.2 | 3TMS |
| Glutamate | 9.405 | 3TMS |
| Ornithine 1 | 9.445 | 3TMS |
| Phenylalanine | 9.56 | 2TMS |
| Isocitric acid | 9.76 | 2TMS |
| Asparagine | 9.79 | 3TMS |
| Taurine | 9.825 | 2TMS |
| Ribose | 9.88 | 5TMS |
| Glutamine 1 | 10.16 | 4TMS |
| Glycerol-3-Phosphate | 10.33 | 4TMS |
| Glutamine 2 | 10.365 | 3TMS |
| 2-Acetylpyridine | 10.435 | TMS |
| O-Phosphorylethanolamine | 10.465 | 4TMS |
| Hypoxanthine | 10.57 | 2TMS |
| Ornithine 2 | 10.635 | 4TMS |
| Citrate | 10.64 | 4TMS |
| 2-Ketoglutarate | 10.845 | MEOX, 2TMS |
| Fructose | 10.98 | MEOX, 2TMS |
| Glucose 1 | 11.06 | MEOX, 2TMS |
| Lysine | 11.165 | 4TMS |
| Histidine | 11.165 | 4TMS |
| Tyrosine | 11.265 | 3TMS |
| Glucitol | 11.34 | 6TMS |
| Mannonic acid | 11.395 | 6TMS |
| Sebic acid | 11.42 | 2TMS |
| Gluconic Acid | 11.485 | 6TMS |
| Pantothenic acid | 11.54 | 3TMS |
| Palmitoleic acid | 11.615 | TMS |
| Xanthine | 11.675 | 3TMS |
| Palmitic acid | 11.7 | 4TMS |
| Octadecadienoate 1 | 11.965 | TMS |
| D-Ribose-5-phosphate | 12.05 | 5TMS |
| Uric acid | 12.075 | 4TMS |
| Inositol | 12.095 | 6TMS |
| 2-Glycerophosphate | 12.135 | 5TMS |
| D-Ribose-5-phosphate | 12.205 | 5TMS |
| Glucose 2 | 12.31 | 5TMS |
| Octadecadienoate 2 | 12.5 | TMS |
| Oleic acid | 12.535 | TMS |
| Octadecadienoate 3 | 12.61 | TMS |
| Tryptophan | 12.65 | 3TMS |
| Arachidonic acid 1 | 12.735 | TMS |
| Fructose-6-Phosphate | 13.035 | 6TMS |
| D-Mannose-6-Phosphate | 13.09 | 6TMS |
| Arachidonic acid 2 | 13.195 | TMS |
| Glucose-6-Phosphate | 13.23 | 6TMS |
| Arachidonic acid 2 | 13.275 | TMS |
| Oleamide | 13.36 | TMS |
| Inositol-3-Phosphate | 13.565 | 7TMS |
| Uridine | 13.595 | 3TMS |
| Arachidonic acid 3 | 13.94 | TMS |
| Docosahexaenoic acid | 13.975 | TMS |
| Arachidonic acid 4 | 14 | TMS |
| Inosine | 14.09 | 4TMS |
| D-Sedoheptulose-7-Phosphate | 14.13 | 6TMS |
| Adenosine | 14.35 | 4TMS |
| Cytidine | 14.605 | 4TMS |
| D-Galactofuranose | 14.82 | 6TMS |
| Celloblose | 14.865 | 2TMS |
| Maltose | 15.13 | 8TMS |
| Cholesterol | 17.53 | TMS |

**Table S2. The perturbed cardiac tissue metabolites induced by high-altitude hypoxia and the regulatory effect of drugs on the metabolites**

| **Metabolic pathways/metabolites** | L-carnitine group | |  | Trimetazidine group | |  | L-carnitine+ Trimetazidine group | |
| --- | --- | --- | --- | --- | --- | --- | --- | --- |
|  | Regulate | Significant |  | Regulate | Significant |  | Regulate | Significant |
| **Amino acids metabolism** |  |  |  |  |  |  |  |  |
| alanine, proline, serine, threonine, tryptophan, asparagine, lysine, tyrosine | ↓ | * |  | ↓ | * |  | ± | ／ |
| taurine | ↑ | ／ |  | ↓ | ／ |  | ↓ | ／ |
| **Branched chain amino acid** |  |  |  |  |  |  |  |  |
| valine, leucine, isoleucine | ↓ | * |  | ↓ | * |  | ↑ | * |
| **Fatty acids** |  |  |  |  |  |  |  |  |
| oleic acid | ↓ | * |  | ± | ／ |  | ↓ | * |
| octadecanoic acid, arachidonic acid, docosahexaenoic acid | ↓ | * |  | ± | ／ |  | ↓ | * |
| glyceric acid | ↓ | * |  | ± | ／ |  | ↓ | * |
| **Ketone** |  |  |  |  |  |  |  |  |
| 3-hydroxybutyrate | ↑ | ／ |  | ↑ | * |  | ↓ | * |
| **Carbohydrates and glycolysis** |  |  |  |  |  |  |  |  |
| glucose, fructose, galactose, mannose | ± | ／ |  | ↓ | * |  | ↓ | * |
| lactate | ± | ／ |  | ± | ／ |  | ↓ | ／ |
| pyruvate | ↓ | ／ |  | ± | ／ |  | ↓ | ／ |
| **TCAs** |  |  |  |  |  |  |  |  |
| citrate | ± | ／ |  | ↑ | * |  | ↓ | * |
| malate | ± | ／ |  | ↓ | * |  | ↓ | * |
| fumaric acid | ± | ／ |  | ± | ／ |  | ↓ | * |
| α-Ketoglutaric acid | ± | ／ |  | ± | ／ |  | ↑ | ／ |
| succinic acid | ± | ／ |  | ± | ／ |  | ↓ | * |

The regulatory tendency and statistic analysis relative to the control group.

↓: Down-regulated by at least 20%, respectively;

↑: Up-regulated by at least 20%, respectively; ±: marginally regulated without statistic significance.

*, *p*<0.05 (one way ANOVA). ／: *p*>0.05 (one way ANOVA).
